# NMDA Receptors Mediate Stimulus-Timing-Dependent Plasticity and Neural Synchrony in the Dorsal Cochlear Nucleus

**DOI:** 10.3389/fncir.2015.00075

**Published:** 2015-11-20

**Authors:** Roxana A. Stefanescu, Susan E. Shore

**Affiliations:** ^1^Department of Otolaryngology, Kresge Hearing Research Institute, University of MichiganAnn Arbor, MI, USA; ^2^Department of Molecular and Integrative Physiology, University of Michigan Medical SchoolAnn Arbor, MI, USA; ^3^Department of Biomedical Engineering, University of MichiganAnn Arbor, MI, USA

**Keywords:** NMDA receptors, dorsal cochlear nucleus, neural plasticity, stimulus-timing-dependent plasticity, neural synchrony, tinnitus, mechanisms of neural plasticity

## Abstract

Auditory information relayed by auditory nerve fibers and somatosensory information relayed by granule cell parallel fibers converge on the fusiform cells (FCs) of the dorsal cochlear nucleus, the first brain station of the auditory pathway. *In vitro*, parallel fiber synapses on FCs exhibit spike-timing-dependent plasticity with Hebbian learning rules, partially mediated by the NMDA receptor (NMDAr). Well-timed bimodal auditory-somatosensory stimulation, *in vivo* equivalent of spike-timing-dependent plasticity, can induce stimulus-timing-dependent plasticity (StTDP) of the FCs spontaneous and tone-evoked firing rates. In healthy guinea pigs, the resulting distribution of StTDP learning rules across a FC neural population is dominated by a Hebbian profile while anti-Hebbian, suppressive and enhancing LRs are less frequent. In this study, we investigate *in vivo*, the NMDAr contribution to FC baseline activity and long term plasticity. We find that blocking the NMDAr decreases the synchronization of FC- spontaneous activity and mediates differential modulation of FC rate-level functions such that low, and high threshold units are more likely to increase, and decrease, respectively, their maximum amplitudes. Three significant alterations in mean learning-rule profiles were identified: transitions from an initial Hebbian profile towards (1) an anti-Hebbian; (2) a suppressive profile; and (3) transitions from an anti-Hebbian to a Hebbian profile. FC units preserving their learning rules showed instead, NMDAr-dependent plasticity to unimodal acoustic stimulation, with persistent depression of tone-evoked responses changing to persistent enhancement following the NMDAr antagonist. These results reveal a crucial role of the NMDAr in mediating FC baseline activity and long-term plasticity which have important implications for signal processing and auditory pathologies related to maladaptive plasticity of dorsal cochlear nucleus circuitry.

## Introduction

The dorsal cochlear nucleus (DCN) is the first auditory station in the central nervous system that integrates multisensory information. Multimodal signal processing is critical for achieving a precise representation of the environment. For instance, by combining auditory cues with somatosensory input about the position and movement of the head, DCN provides more accurate sound localization (Sutherland et al., 1998; May, 2000). Because DCN neural circuitry shares many characteristics with cerebellar-like structures (Devor, 2000; Oertel and Young, 2004), a functional similarity has been proposed among these structures. In particular, adaptive processes were proposed to reduce the responses of principal cells to predictable stimuli such as the animal’s self-generated signals and movements (Fujita, 1982; Oertel and Young, 2004; Bell et al., 2008; Roberts and Portfors, 2008; Dean et al., 2010; Requarth and Sawtell, 2011). Because DCN has multiple molecular mechanisms mediating its plasticity (Fujino and Oertel, 2003; Tzounopoulos et al., 2004; Zhao and Tzounopoulos, 2011), elucidating these mechanisms can provide unique insights into the functions of the DCN and other cerebellar-like circuits.

The fusiform cells (FCs) of the DCN integrate cochlear input from auditory nerve fibers (ANFs) that synapse on their basal dendrites, with somatosensory input relayed by the parallel fiber (PF) axons of CN granule cells that synapse on their apical dendrites. FC activity is modulated by inhibition from cartwheel cells (CWC) and superficial stellate cells in the DCN molecular layer, and vertical cells in the deep DCN. FCs provide the main output from DCN to the inferior colliculus (Oertel and Golding, 1997; Young and Davis, 2002). Early studies reported bidirectional plasticity for the PF-FC and PF-CWC synapses but not at the ANF-FC synapses (Fujino and Oertel, 2003). Furthermore, *in vitro* investigations of spike-timing dependent plasticity (SpTDP) revealed Hebbian plasticity at the PF-FC synapses but anti-Hebbian plasticity at PF-CWC synapses (Tzounopoulos et al., 2004). These so called plasticity “learning rules” (LRs) are mediated by a complex set of mechanisms of which the NMDA receptor (NMDAr), which is robustly expressed at the ANF-FC and PF-FC and PF-CWC synapses (Rubio et al., 2014), is a critical component.

Blocking the NMDAr *in vitro* prevents induction of long term potentiation (LTP) in FCs (Tzounopoulos et al., 2007). This observation is consistent with findings in hippocampus (Collingridge et al., 1983; Davis et al., 1992; Kamiya et al., 1993; Murphy et al., 1997) and a majority of other brain areas (Hunt and Castillo, 2012), establishing a central role of the NMDAr in synaptic plasticity. Furthermore, the unique circuitry and receptor distribution in the DCN promotes a robust interaction of the NMDAr with muscarinic and endocannabinoid signaling pathways in both PF-FC and PF-CWC synapses. For instance, an NMDAr mediated increase in intracellular Ca^2+^ induces Hebbian LTP but anti-Hebbian long term depression (LTD) when these events are coordinated with simultaneous activation of M1/M3 muscarinic acetylcholine receptors (mAChRs; Zhao and Tzounopoulos, 2011). In CWCs, blocking the endocannabinoid receptor, CB1, which is more abundant at PF-CWC post-synaptic sites, induces (LTP) for stimulation protocols that would otherwise induce LTD. Remarkably, the signaling cascades leading to both LTP and LTD are initiated in the postsynaptic cell by a rise in NMDAr-mediated Ca^2+^. Activation of the endocannabinoid receptor at the PF-CWC synapses then secures the anti-Hebbian LR in CWC (Tzounopoulos et al., 2007).

In general, SpTDP- inducing stimulation not only modulates synaptic strength but can also alter intrinsic neural excitability (Desai, 2003; Belmeguenai et al., 2010; Debanne and Poo, 2010) and therefore Spontaneous activity and stimulus-driven neural spiking patterns (Turrigiano et al., 1994; Mahon et al., 2003; Phoka et al., 2012).

The coordinated interaction between these diverse mechanisms is likely to have complex effects in modulating *in vivo* plasticity and associated functional characteristics of DCN circuitry. Indeed, recent *in vivo* investigations of neural plasticity demonstrated that LRs for tone-evoked and spontaneous activity can be induced by a bimodal stimulation (BM) protocol in which auditory stimulation is delivered in temporal proximity with somatosensory stimulation of the spinal trigeminal nucleus (Sp5) at various stimulus onset differences, i.e., bimodal intervals (BIs; Koehler and Shore, 2013a,b). Consistent with the SpTDP LRs, a majority of DCN FCs showed stimulus-timing-dependent plasticity (StTDP), Hebbian LRs. However, other types of LRs including anti-Hebbian, suppressive and enhancing were also found in FCs *in vivo*. Interestingly, the type of LRs correlated with the degree of FC inhibition (as quantified by FC receptive field maps) suggesting a contribution of the balance of the excitatory/inhibitory synaptic input in shaping FC plasticity (Koehler and Shore, 2013b).

This rich repertoire of bimodal plastic modifications of FC activity is critically important in adaptive filtering properties of the DCN, including those involved for sound-localization (Oertel and Young, 2004; Roberts and Portfors, 2008). Furthermore, the distribution of LRs in DCN are altered by noise exposure and tinnitus, thereby changing the population LR from a Hebbian profile in sham-exposed animals to an anti-Hebbian profile in tinnitus animals (Koehler and Shore, 2013a). Elucidating the contribution of specific receptors to *in vivo* DCN plasticity will therefore augment our understanding of DCN function and indicate possible contributors to the alterations associated with tinnitus pathology.

In this article, we investigate *in vivo*, the effects of the NMDAr on baseline spiking activity and StTDP of DCN FCs. We find that blocking the NMDAr reduces the synchrony of spontaneous firing between FCs and alters FC rate-level functions (RLFs) by increasing the maximum amplitude of low-threshold FCs and decreasing the maximum amplitude of high threshold FCs, respectively. In addition, tone-evoked StTDP LRs change from primarily Hebbian to anti-Hebbian or suppressive profiles and from anti-Hebbian to Hebbian profiles after blocking the NMDAr. LR-preserving units showed NMDAr-dependent plasticity to unimodal acoustic (UA) stimulation, whereby persistent suppression changed to persistent enhancement of FC tone-evoked responses following NMDAr antagonist. Together, these results suggest that the NMDAr contributes significantly to FC activity and plasticity with implications for signal processing and auditory pathologies characterized by maladaptive plasticity in the DCN (Wu et al., 2015).

## Materials and Methods

Five normal-hearing guinea pigs (Elm Hill colony, Ann Arbor, MI, USA; 300–400g) were used in this study. All procedures were performed in accordance with the *National Guidelines for the Use and Care of Laboratory Animals* (NIH Publications No. 80–23) and the guidelines and approval by the University Committee on Use and Care of Animals of the University of Michigan.

### Auditory Brainstem Response Recordings

Guinea pigs were anesthetized (see “Surgical Methods” Section) and auditory brainstem response (ABR) thresholds were measured immediately before unit recordings. ABRs were collected using BioSigRP software and RA4LI/RX8/RZ2 hardware [Tucker-Davis Technologies (TDT), Alachua, FL, USA]. Stimuli were 10 ms tone pips (2 ms ramp, 11 stimuli/s) from 4–20 kHz in step in 2 kHz steps, starting at 90 dB SPL and decrementing in 10 dB steps with 512 repetitions per level. ABR waveforms were visually inspected across levels, and the threshold was determined for each frequency as the lowest sound level for which the ABR waves were distinguishable by eye from background noise. All the animals considered in this study showed normal hearing thresholds (Djalilian and Cody, 1973; Ingham et al., 1998) in the range 0–30 dB across all frequencies tested.

### DCN Unit Recordings

#### Surgical Methods

Guinea pigs were anesthetized with subcutaneous injections of ketamine (40 mg/Kg; Putney, Portland, OR, USA) and xylazine (10 mg/Kg; Lloyd) followed by local subcutaneous injections of lidocaine (4 mg/Kg) at the incision sites. The animals’ heads were rigidly fixed in a Kopf stereotaxic frame with hollow ear bars placed in their ear canals and secured with a bite bar. Their eyes were protected with ophthalmic ointment and rectal temperature maintained at 38 ± 0.5°C using a temperature-controlled heating pad and a rectal probe. A rostral-caudal incision was made and the skin retracted to reveal the skull.

#### Drug-Delivery of NMDAr Antagonist to DCN

**Figure 1:**
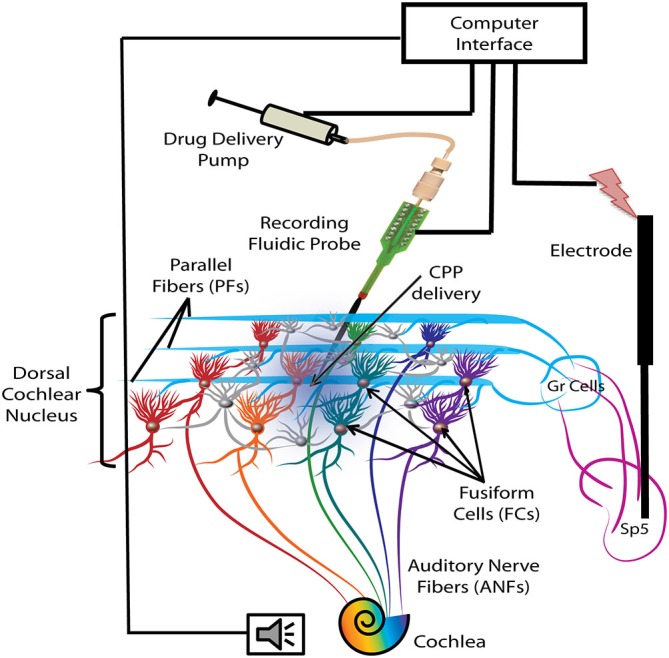
**Schematic representation of the experimental protocol.** Electrical stimulation was delivered with a bipolar electrode implanted in the Sp5 brainstem nucleus. Auditory stimulation activates the auditory nerve fibers (ANFs) of the cochlea and the FCs through direct synapses on their basal dendrites. Stimulation of Sp5 activates the granule cell (Gr) axons, the parallel fibers (PFs), which synapse on the apical dendrites of the FCs. FC activity was recorded with a fluidic probe (NeuroNexus) placed stereotaxically into the dorsal cochlear nucleus (DCN) FC layer. The probe was connected to a computer-controlled drug delivery system to deliver the NMDA receptor (NMDAr) blocker solution (CPP 2 μl, 100 μM, 100 nl/min) directly into the FC layer (blue gradient color).

A 1-shank, 16 electrode neuroprobe with integrated drug-delivery interface (D16, NeuroNexus, Ann Arbor, MI, USA) was connected to a 10 μl syringe loaded with a 100 μM solution of the NMDAr antagonist, CPP (3-(*R*)-2-Carboxypiperazin-4-yl)-propyl-1-phosphonic acid, Tocris Bioscience, Bristol, UK) and fixated in a digitally-controlled electric pump (UMP3 microsyringe injector with Micro4 controller, World Precision Instruments, Inc, Sarasota, FL, USA). This system allows simultaneous recording of neural activity and drug delivery as described and validated in a previous study (Rohatgi et al., 2009). After craniotomy and durectomy, the probe was inclined at 25° and inserted stereotaxically into the left DCN, 3 mm lateral to the midline, 4 mm caudal to the interaural 0 and 5.5–6 mm depth (see Figure 1 for a schematic representation). Additional displacements in depth were performed in steps of 200 μm until a majority of the recording sites were positioned in the fusiform layer as indicated by extracellular robust responses to noise stimuli (1000 trials, each of 50 ms noise stimuli with 5 ms ramp) and characteristic temporal responses to tones for FCs were obtained. To assess the effects of blocking the NMDArs, 2 μl of the CPP 100 μM solution was delivered into the FC layer with a velocity of 100 nl/min (optimal for this configuration; Rohatgi et al., 2009) after initial assessments on baseline FC activity, FC synchrony of SFRs and stSTDP.

#### Electrical Stimulation of Sp5

A second concentric bipolar stimulating electrode (FHC, Bowdoin) was dipped in Flurogold and stereotaxically placed in the left Sp5 (Sp5; 0.28 ± 0.03 cm lateral from midline, 0.25 ± 0.02 cm caudal from transverse sinus, 0.9 ± 0.1 cm below the surface of the cerebellum). The location of the stimulating electrode was confirmed post mortem by histological localization of the Fluorogold stain.

#### Assessment of Responses to Auditory and Somatosensory Stimulation

The threshold and best frequency (BF) was assessed for each FC unit from its receptive field, which was constructed by counting the spikes produced in response of a total of 7600 tone bursts over an intensity range of 0–90 dB (in steps of 5 dB) and a frequency range of 100 Hz–24 kHz (in 0.2 octave steps). Peristimulus time histograms (PSTHs), tone and noise RLFs were collected to determine the unit type based on previously described criteria (Evans and Nelson, 1973; Young and Brownell, 1976; Young, 1980; Rhode et al., 1983; Stabler et al., 1996; Ding and Voigt, 1997). FCs were identified as units with pause build-up (P–B), build-up (B) and choppers (C) based on their PSTHs in response to BF tones at 20 dBSL (1000 trials, each of 50 ms, 5 ms ramp) receptive field types and BF tone and noise RLFs characteristic of FCs as in previous studies (Young and Brownell, 1976; Stabler et al., 1996; Dehmel et al., 2012; Koehler and Shore, 2013a,b). Units with atypical PSTHs or receptive field responses inconsistent with fusiform cell classification were discarded from analysis. The tones were generated using OpenEX and Rx8 DSP systems (TDT) and delivered to the ear canal via a hollow left ear bar by an attached shielded speaker (DT770; Beyer) driven by a HB7 amplifier (TDT). Sound levels were adjusted with a programmable attenuator (PA5; TDT) previously calibrated for equal levels at frequencies between 100 Hz and 24 kHz.

Sp5 stimulation was performed using biphasic current pulses (100 μs/phase) delivered at 1 kHz through a bipolar electrode. The current amplitude was set to a level that just evoked spikes in FCs and did not elicit any movement artifact (between 50 and 70 μA).

#### Synchrony of Spontaneous Firing Activity

**Figure 2:**
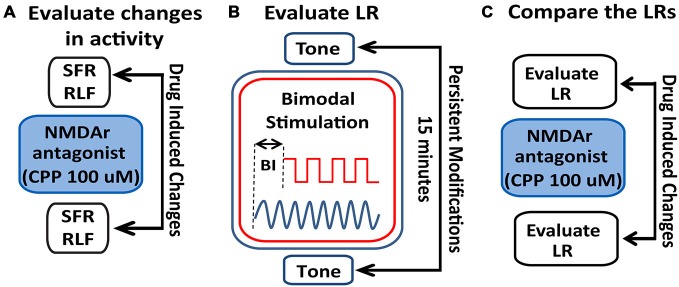
**Schematic illustration of the stimulation protocol and evaluation of LRs changes following NMDAr blockage. (A)** Spontaneous activity (SFR) and tone-evoked rate-level functions (RLFs) were evaluated before the bimodal stimulation (BM) protocol and before and after CPP delivery (2 μl, 100 μM) to assess differences in FC activity mediated by the NMDAr. **(B)** To determine a LR, tone-evoked firing rates are evaluated before and 15 min after BM at various bimodal intervals (BIs). **(C)** Effects of the NMDAr antagonist, CPP, are then evaluated by comparing the LRs obtained before and after drug delivery.

Spontaneous activity was recorded continuously for 2.5 min before the BM protocol and before and after CPP delivery (Figure 2A). Synchrony of spiking between units was evaluated as described in Voigt and Young (1988, 1990) and Eggermont (1992). To eliminate possible artificial correlations, common spikes occurring within ±150 μs on different electrodes were eliminated (Brody, 1999). Units with mean spontaneous firing rates of less than 1 Hz were excluded from further analysis. The peak cross-correlation coefficients *ρ*(τ) were computed according to the equation below Abeles (1982), Eggermont (1992) for each time lag (τ) and for each pairwise combination of spike trains:

$$\rho (\tau )=\frac{R_{AB}(\tau )-E}{{\left(N_{A}N_{B}\right)}^{0.5}}$$

where, R_AB_ is the unbiased cross-correlation of spike trains A and B with N_A_ and N_B_ number of spikes, $E=\frac{N_{A}N_{B}}{N}$ is the expectancy of spike train coincidence, under the assumption of independence, and *N* is the number of bins. The bin size was 0.3 ms (Voigt and Young, 1990). Only time lags between −20 and +20 ms were analyzed. Unit pairs exhibited synchrony when the ρ(τ) exceeded four standard deviations from the mean ρ across all time lags considered. Each unit was paired with another single unit and all possible combinations were evaluated. Synchronization between any two units was reported just once (synchronization between *A* and *B* is equal with the synchronization between *B* and *A*).

#### Rate Level Functions

**Figure 3:**
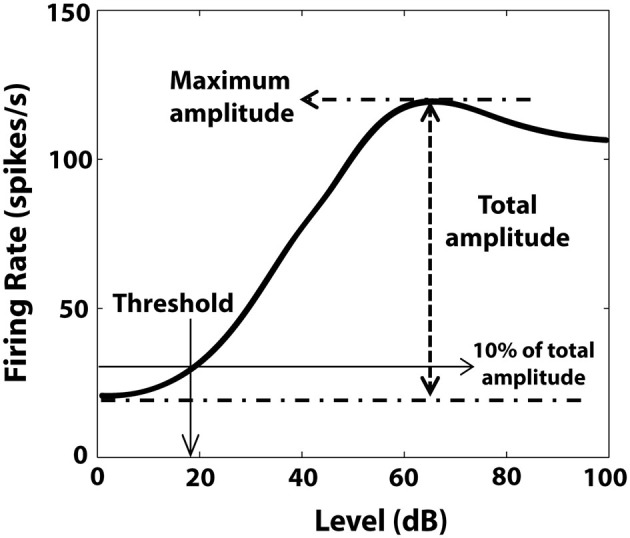
**Illustration of RLF evaluation.** Maximum amplitude of the RLF was determined by the peak firing rate value of the RLF. The threshold was the level corresponding to 10% of the total amplitude, defined by the difference between maximum and minimum RLF firing rate values.

RLFs were evaluated before the BM protocol and before and after CPP delivery. BF tones (50 ms tone/trial, 4.5 ms rise/fall time) were varied pseudo-randomly in level, from 0–85 dB in 5 dB steps (Figure 2A). The number of spikes elicited by each tone was counted and the averaged RLFs were constructed from 50 presentations at each level of each stimulus. The RLFs were than smoothed (using the function smooth in Matlab) and the RLF maximum amplitude was determined by the peak firing rate value of the RLF (Figure 3). The threshold was determined at 10% of the total RLF amplitude (the difference between the maximum and minimum RLF firing rate values).

#### StTDP Assessment

StTDP was assessed *in vivo* using a bimodal StTDP-inducing protocol previously described in Koehler and Shore (2013b). Briefly, BF tone-evoked responses were recorded before and 15 min after BM: the BM consisted of 300 trials (200 ms/trial) of 50 ms BF tones and Sp5 pulses (bipolar, 100 μs/phase) presented at 1 Hz, intertone interval 100 ms. The onset difference between tone and Sp5 stimulation defines the BI. Negative BI values indicate sound leading Sp5 stimulation, while positive BI values indicate Sp5 leading sound stimulation (Figure 2B). Following the BM protocol and LR-assessment, CPP was delivered and the LRs reassessed. LRs obtained before and after CPP delivery were then compared (Figure 2C).

### Data Analysis

#### Spike Detection and Sorting

Waveforms from each electrode site were digitized using a PZ2 preamplifier (*F*s = 12 kHz, Tucker Davis Technologies, TDT) and band-passed filtered (300 Hz–3 kHz). A voltage threshold of 2.5 standard deviations from the background noise was used for online spike detection (RZ2 module, TDT) and the timestamps and waveform snippets were saved on a PC. Offline Sorter software (Plexon Inc., Dallas, TX, USA) was used to manually cluster the waveforms based on their similarity in the first three principal components. The clusters representing single-unit activity were tracked for consistency across recordings performed during the StTDP protocol. The time-stamps of the sorted units were imported in MATLAB for further analysis.

#### Statistical Analysis

One-way ANOVA (with statistical significance set at 0.05) was used to determine statistical significance of changes in the strength of synchronization and changes in RLFs following CPP delivery. Two-way ANOVA with drug and BI as factors was used to assess the effects of CPP on units changing and preserving their LRs, respectively. The statistical significance was set at 0.05.

## Results

### Effects of NMDAr Antagonist on FC Activity

#### NMDAr Antagonist Decreases Synchrony of Spontaneous Spikes Across Fusiform Cells

**Figure 4:**
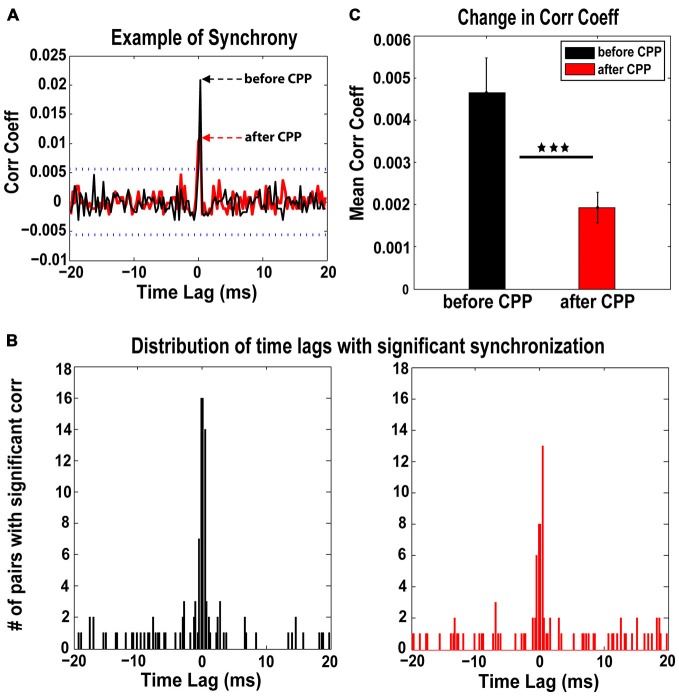
**The NMDAr antagoinist, CPP, alters synchronization of FC spontaneous activity. (A)** Representative example of cross-correlation histogram of spontaneous spikes for a pair of FCs evaluated before (black) and after (red) CPP delivery. The 95% confidence boundaries are indicated by the dotted lines. Note the decreased coefficient of correlation at time lag of 0 ms following CPP. **(B)** The distribution of significant time lags of synchronization before CPP (left panel, black) is centered at 0 ± 0.5 ms and does not change significantly (Kolmogorov-Smirnov 2-sample test: *p* = 0.13) following CPP delivery (right panel, red). **(C)** In contrast, the strength of synchronization indicated by the mean correlation coefficient decreases significantly following CPP (one-way ANOVA, *F*_(1)_ = 8.98, *p* = 0.003). Statistical significance is indicated by black stars.

Synchrony of FC spontaneous spikes and the corresponding time lags were assessed before and after CPP using a previously established measure of spike train correlations between unit pairs in the auditory cortex and DCN (Voigt and Young, 1988, 1990; Norena and Eggermont, 2003). Across the neural population analyzed (98 single units in five animals), 46 distinct pairs of units showed significant synchronization both before and after CPP (see Figure 4A for a representative example). For each pair, we assessed the strength of synchrony (the correlation coefficient that exceeded the significance threshold) and its corresponding time lags. The time-lag distribution before CPP was centered at 0 ± 0.5 ms (Figure 4B, left panel) and did not change significantly (Kolmogorov-Smirnov 2-sample test, *p* = 0.13) following CPP (Figure 4B, right panel). However, the synchronization strength decreased significantly (one-way ANOVA, *F*_(1)_ = 8.98, *p* = 0.003) across the fusiform cell neural population following CPP (Figure 4C).

#### NMDAr Antagonist Differentially Modulates RLF Maximum Amplitude

**Figure 5:**
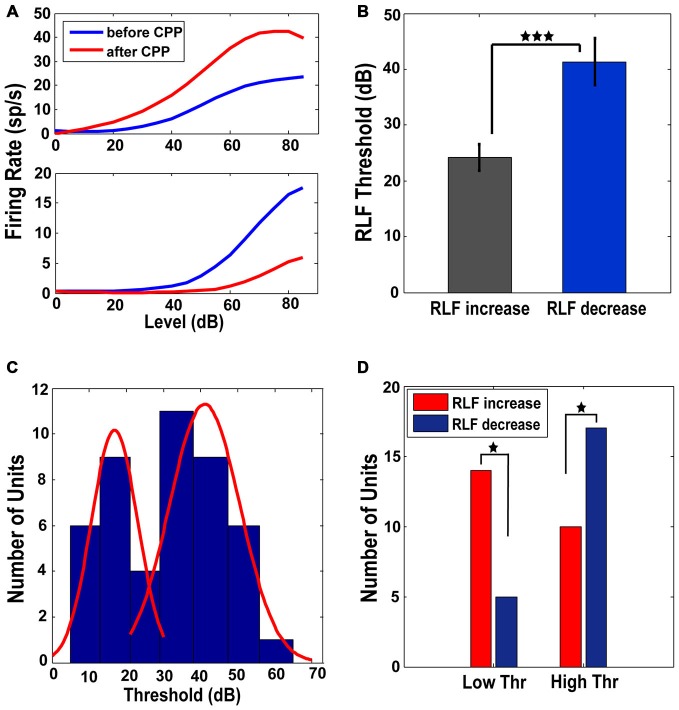
**Low and high threshold RLFs show opposite changes following CPP. (A)** Representative RLFs for two different FCs that show increases (upper panel) and decreases (lower panel), respectively, following CPP delivery. **(B)** Units that show increased RLFs have statistically significantly lower thresholds than the units showing decreases of their RLFs. **(C)** The distribution of thresholds across the FC population has a bimodal profile (blue histogram) with a border at 30 dB, best fitted by a 2-gaussian mixture model with individual distributions (red lines) centered at 17 dB (standard deviation of 6.3 dB) and 41 dB (standard deviation of 9.5 dB). **(D)** A significant number of units with low thresholds (<30 dB) show increases in their RLF maximum amplitude (red) while a significant number of units with high thresholds (≥30 dB) show a decrease (blue) in their RLF maximum amplitude following CPP delivery. The statistical significance is indicated in **(B,D)** by black stars.

RLFs were collected at BF before and after CPP. Only the units (*n* = 46 or 47% of the total units analyzed) in which the RLF functions after CPP were significantly different (see “Materials and Methods” Section) from those evaluated before CPP were considered for further analysis. These units showed two types of changes. One population (N1 = 24 or 52%) increased the maximum amplitude of their RLFs (one-way ANOVA, *F*_(1)_ = 10.73, *p* = 0.002) while the rest (N2 = 22 or 48% of units) decreased their RLF maximum amplitude (one-way ANOVA, *F*_(1)_ = 6.69, *p* = 0.013; see Figure 5A for representative examples). The units in with increases in their RLF maximum amplitudes had significantly lower thresholds (one-way ANOVA, *F*_(1)_ = 11.4, *p* = 0.0004) than the units showing a decrease in their RLF maximum amplitudes (Figure 5B). The histogram of the initial threshold distribution showed a bimodal profile with a border at 30 dB (Figure 5C) that was best fit by a 2-gaussian mixture model with individual distributions (Figure 5C, red lines) centered at 17 dB (standard deviation of 6.3 dB) and 41 dB (standard deviation of 9.5 dB) respectively. This distribution remained bimodal with no significant alterations following CPP delivery (Kolmogorov-Smirnov test, *p* = 0.624). Therefore, we divided the neural population in two subgroups of low threshold (lower than 30 dB) and high threshold (higher or equal to 30 dB) units, respectively. A significant number of low threshold units showed an increase in their RLF maximum amplitude while a significant number of units with high threshold showed a decrease in their RLF maximum amplitude following CPP (Fisher exact test, *p* = 0.018; Figure 5D). Thus, FCs with lower thresholds were more likely to increase their RLF maximum amplitudes following CPP, while the units with higher thresholds were more likely to decrease their RLF maximum amplitudes after CPP.

### Effects of NMDAr Antagonist on FC StTDP

#### NMDAr Antagonist Mediates Three Patterns of Transitions in the StTDP induced LRs

**Figure 6:**
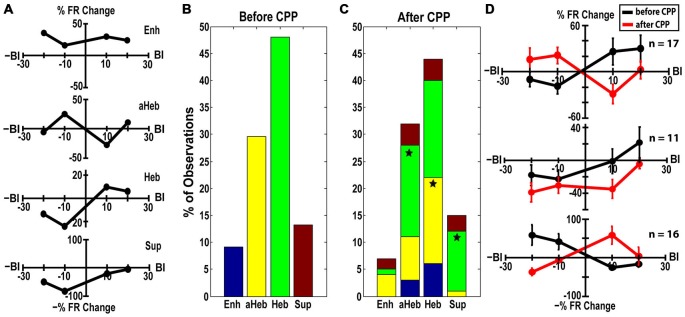
**CPP alters FC LRs. (A)** Representative examples of Hebbian (Heb), anti-Hebbian (aHeb), suppressive (Sup) and enhancing (Enh) LR profiles recorded in individual FC units. **(B)** Before CPP, 48% of units showed Heb LRs (green), 30% aHeb (yellow), 9% Enh (blue) and 13% Sup (red) profiles. **(C)** After CPP, for the FCs with initial Heb LR profiles, a significant number (35%) transitioned to an aHeb profile. The remaining units either preserved their profiles (38%) or showed significant transitions (23% of units) towards suppressive profiles. For units with aHeb initial profiles, a significant number (55%) transitioned towards Heb profiles after CPP delivery; 28% of the units conserved their initial profiles and 14% transitioned towards an enhancing profile. A small number of units with initial Enh or Sup LR profiles also transitioned towards Heb and aHeb profiles following NMDAr antagonist. The color coding is consistent with **(B)**. Statistical significance is indicated by stars. **(D)** Mean LRs before (black) and after (red) CPP in the FC subpopulations displaying three distinct patterns of LR transitions: from Hebbian to anti-Hebbian (top panel; *n* = 17 units) and suppressive profile (middle panel; *n* = 11 units), respectively and from anti-Hebbian to Hebbian profile (bottom panel; *n* = 16 units).

StTDP LRs were evaluated in 98 FCs before and after CPP. LRs were classified into four distinct profiles (Hebbian, anti-Hebbian, enhancing and suppressive; Figure 6A) as previously identified *in vivo* (Koehler and Shore, 2013a,b). Before CPP, 48% of units showed Hebbian LRs, 30% had anti-Hebbian LRs, 9% were enhancing and 13% were suppressive (Figure 6B). Following CPP, the LRs were redistributed (Figure 6C) across the FC population. Three distinct patterns of changes in the LR profiles were identified:

(a) Hebbian LRs change to anti-Hebbian LRs. Of the 47 FCs with initial Heb LRs (Figure 6B, green), 35% transitioned to an aHeb profile (χ^2^ = 27.19, *p* < 0.001) following CPP (Figure 6C, green). The mean LRs of this subpopulation are shown in Figure 6D. A two-way ANOVA reveals significant differences between the two LR profiles for interactions between the factors of BI and drug (*F*_(3)_ = 5.55, *p* = 0.0013).
(b) Hebbian LRs transition towards suppressive LRs. Twenty three percent of units with initial Hebbian LRs (Figure 6B, green), showed transitions to a suppressive profile following CPP (Figure 6C, green, χ^2^ = 13.39, *p* < 0.001). Figure 6D—middle panel shows the mean LRs of this subpopulation. A two-way ANOVA test indicated statistical difference between the two LR profiles with BI (*F*_(3)_ = 3.65, *p* = 0.0159) and drug (*F*_(1)_ = 7.27, *p* = 0.0085) as significant factors.
(c) anti-Hebbian LRs transition to Hebbian LRs. Out of 29 units with initial aHeb LRs (Figure 6B, yellow) a significant number (55%) transitioned towards Heb profile after CPP (χ^2^ = 29.93, *p* < 0.001; Figure 6C, yellow). Two-way ANOVA indicated statistical significant differences between the mean LRs evaluated in this subpopulation (Figure 6D, bottom panel) for interactions between the factors of BI and drug (*F*_(3)_ = 10.15, *p* = 5.2092^−06^).

#### Pause-Buildup and Buildup Units are more Likely to Change Their Respective Hebbian and Anti-Hebbian LRs to other Profiles after CPP

**Figure 7:**
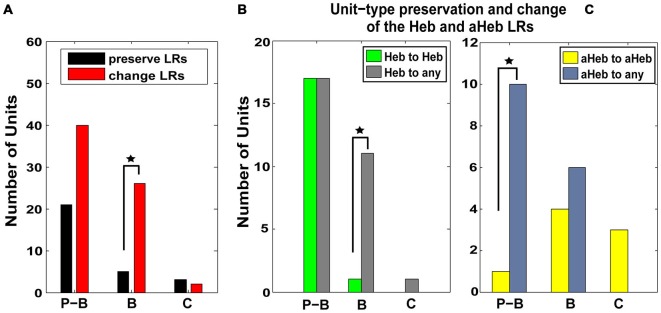
**Pause-buildup and buildup units are more likely to change their respective Hebbian and anti-Hebbian LRs to other profiles. (A)** FCs were classified based on their PSTH into pause-buildup (P–B), buildup (B) and chopper (C). The number of units preserving (black) and changing (red) their LR- profiles, respectively, is displayed for each FC unit type. Both P–Bs and Bs (χ^2^ = 4.1206, *p* = 0.0423) show an increased number of units changing their LRs. **(B)** The number of units that preserve (green) or change (gray) their Hebbian LR profiles following CPP delivery are displayed for each type of PSTH encountered in the neural population. P–B units are more likely to change their initial Hebbian profile to any other type of LR following CPP delivery (χ^2^ = 6.13, *p* = 0.01). **(C)** Similarly, B units are more likely to change their initial anti-Hebbian profile into any other LR type following CPP delivery (χ^2^ = 6.81, *p* = 0.009). Statistical significance is indicate in all panels by black stars.

Units were classified based on their PSTH type (Evans and Nelson, 1973; Young and Brownell, 1976; Young, 1980; Rhode et al., 1983; Stabler et al., 1996; Ding and Voigt, 1997) as pause-buildup (61), buildup (31), and chopper (5) units. Significantly more buildup units (χ^2^ = 4.1206, *p* = 0.0423) and more pause-buildup units changed their LRs profiles (Figure 7A). As the Hebbian and anti-Hebbian profiles constituted a majority (78%) of the LRs across the neural population, we focused the analysis on these patterns. We found that buildup units were significantly more likely to change their Hebbian LRs to any other profile (χ^2^ = 6.13, *p* = 0.01; Figure 7B), while pause buildup units were significantly more likely to change their anti-Hebbian LRs to any other profile (χ^2^ = 6.81, *p* = 0.009; Figure 7C). As the PSTH unit type is determined in part by the expression of A-type K^+^ channels (Kanold and Manis, 1999), this suggest a possible link between the distribution of this channel and the NMDAr mediated FC plasticity.

#### FC Units Preserving their LRs after CPP Exhibit Unimodal Acoustic Plasticity

**Figure 8:**
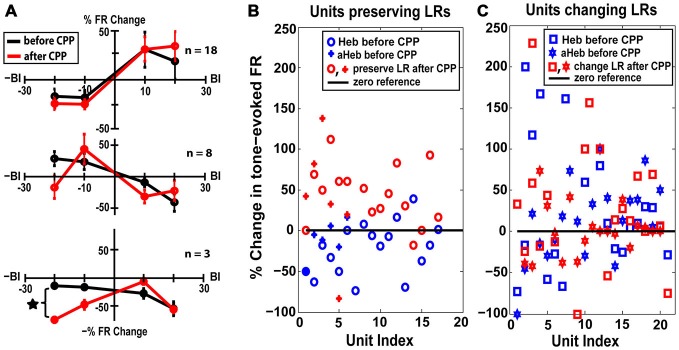
**FC units preserving their LRs exhibit unimodal acoustic (UA) plasticity. (A)** Thirty percent of the FC units preserved their LRs after CPP delivery. Mean LRs are presented before (black) and after (red) CPP in units preserving their Hebbian LRs (upper panel), anti-Hebbian LRs (middle panel) and suppressive LRs (bottom panel). Two-way ANOVA indicated a significant effect for drug (*p* = 0.0416) only for differences in suppressive LRs before and after CPP delivery (black star). **(B)** Percent changes in the tone-evoked firing rate following repeated UA stimulation before (blue) and after (red) CPP delivery. Units that preserve their Hebbian (“o”) or anti-Hebbian (full star) LRs following CPP delivery show distinct plasticity following UA stimulation, i.e., depressing the units’ firing rate before blocking the NMDAr and enhancing it afterwards. **(C)** In contrast, the units that change their LRs (Heb is indicated by “□” and aHeb by empty star) show heterogeneous changes in response to UA stimulation, pattern maintained after CPP delivery.

Thirty eight percent (*n* = 18) of the units with initial Hebbian LRs (χ^2^ = 38.88, *p* < 0.001), 28% (*n* = 8) of the units with initial anti-Hebbian LRs (χ^2^ = 35.85 *p* < 0.001) and a small number (*n* = 3) of units with initial suppressive LRs preserved their LR profiles after CPP (Figure 6C, yellow, green and red). The mean LRs of these FC subgroups are displayed in Figure 8A. For the first two subgroups of Hebbian- and anti-Hebbian- preserving units (Figure 8A, upper and middle panel), respectively, two-way ANOVA showed no statistical significance for drug (*F*_(1)_ = 0.001, *p* = 0.948, *F*_(1)_ = 0.42, *p* = 0.5219) or interaction between drug and BI (*F*_(3)_ = 0.39, *p* = 0.7589, *F*_(3)_ = 2.16, *p* = 0.1042). For the third subgroup, although there was no qualitative change in the FC LR suppressive profiles (Figure 8A, bottom panel), two-way ANOVA showed an effect for drug-bimodal interval interaction (*F*_(2)_ = 4.19, *p* = 0.0416). However, given the limited number of units in this subgroup, the result should be interpreted with caution. To prevent any possible error, these units were eliminated from further analysis.

How do the units that preserve their LR profiles differ from the ones that change their LRs after CPP?

**Figure 9:**
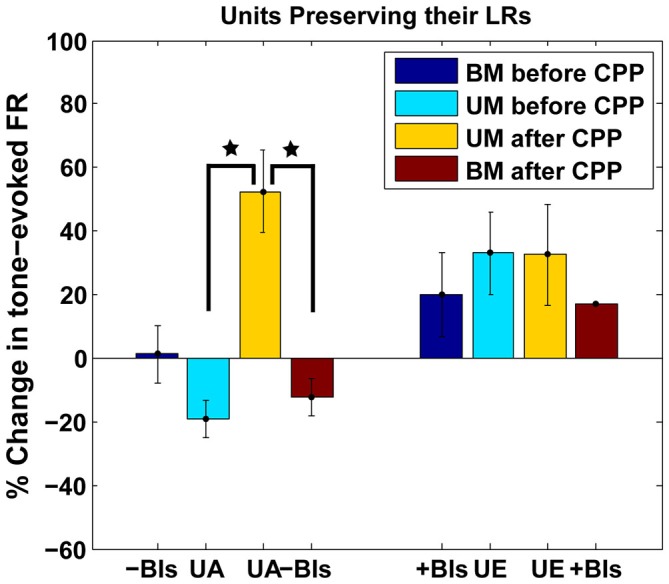
**Comparison of plastic effects induced by bimodal vs. unimodal acoustic and electric stimulation in LR-preserving units.** Mean changes in tone-evoked firing activity after StDP before (blue) and after (red) CPP delivery were evaluated in units preserving their LRs following UA (UA) or electric (UE) stimulation and compared with the changes induced by BM for positive (+BIs) and negative (−BIs) BIs. Blocking NMDAr significantly altered (one-way ANOVA, *F*_(1)_ = 22.33, *p* = 2.37e^−05^) the changes in FC tone-evoked responses following UA stimulation but had no effects on the changes induced by UE stimulation. The changes following UA stimulation were significantly stronger than the ones induced by BM (one-way ANOVA, *F*_(1)_ = 19.56, *p* = 5.91e^−05^). The statistical significance is indicated in the figure by black stars.

The LR-preserving units (Figure 8B) but not the units changing their LRs (Figure 8C) showed consistent suppressive plasticity in response to unimodal acoustic stimulation (UA) that changed significantly (one-way ANOVA, *F*_(1)_ = 22.33, *p* = 2.37e^−05^) to an enhancing plasticity following CPP delivery. The UA induced plasticity induced following CPP delivery was significantly stronger (one-way ANOVA, *F*_(1)_ = 19.56, *p* = 5.91e^−05^) than the plasticity induced by BM for negative BIs (for which the sound precedes the SP5 electrical stimulation; Figure 9). In contrast to effects induced by UA, the LR-preserving units showed an enhancement of their tone-evoked responses following unimodal electric stimulation (UE) that remained unchanged following CPP delivery and not significantly different (one-way ANOVA, *p* = 0.2864) from the enhancement induced by BM for positive BIs (for which the SP5 electrical stimulation precedes the sound; Figure 9). Furthermore, the LR-preserving units showed a significant correlation (*R*^2^) between their spontaneous rates and their coefficients of variation (Heb: *R*^2^ = 0.48; *p* = 0.04, aHeb: *R*^2^ = 0.81, *p* = 0.015) which might suggest a possible contribution of these units to temporal coding (Song et al., 2000). These results indicate that there is a subpopulation of FCs for which the StTDP plasticity, presumably mediated by SpTDP at their PF-FC synapses, is less dependent on the NMDAr. Instead, these units show an unexpected NMDA-mediated plasticity to UA stimulation perhaps due to specific properties of the NMDAr expressed at the ANF-FC synapse.

## Discussion

In this study, we analyzed the changes in FC activity and StTDP following NMDAr antagonist delivery *in vivo*. We demonstrated that blocking NMDArs with CPP reduced synchronization of spontaneous activity and differentially affected fusiform cell RLFs. There were also significant changes in the FCs StTDP including transitions from Hebbian to anti-Hebbian and suppressive LR profiles and from anti-Hebbian to Hebbian profiles. We discuss below the implications of these findings for auditory-multisensory processing.

### Effects of NMDAr Antagonist on FCs Activity

#### Decreased Synchronization of Spontaneous Firing

Blocking the NMDAr leads to decreased synchronization of FC spontaneous activity but not to a significant change in the distribution of time lags at which the synchronization is achieved (Figure 3). The importance of the NMDAr in mediating the synchronization of neural activity has been extensively studied in cortical areas and hippocampus, especially in relationship to schizophrenia pathology (Coyle et al., 2003; Kristiansen et al., 2007; Schwartz et al., 2012; Kane, 2015). In this context, NMDAr hypofunction decreases GABA-ergic activity in the prefrontal cortex, leading to a delayed increase in pyramidal neuron firing rates (Homayoun and Moghaddam, 2007) and disrupted spontaneous synchronization (Kargieman et al., 2007; Kirli et al., 2014). It is possible that similar mechanisms are available in the DCN, which could explain the decreased synchronization of FC activity reported in the current study.

Previous studies in auditory cortex demonstrated increased synchronization of spontaneous activity after acoustic trauma (Norena and Eggermont, 2003; Seki and Eggermont, 2003). Consequently, enhanced synchronization was proposed as an underlying mechanism for tinnitus, the pathology of phantom sound perception (Weisz et al., 2007; Eggermont and Tass, 2015). It is possible this feature of abnormal neural activity occurs at multiple stations of the auditory pathway. Indeed, following noise exposure and tinnitus, enhanced spontaneous activity has been reported in the DCN (Kaltenbach and Afman, 2000; Kaltenbach et al., 2004; Dehmel et al., 2012; Koehler and Shore, 2013a). As spontaneous firing rates can correlate with the degree of synchronization (Norena and Eggermont, 2003), it is possible that enhanced synchronization also occurs in DCN in relation to tinnitus, probably mediated by maladaptive auditory-somatosensory plasticity (Wu et al., 2015). Our study demonstrates that the NMDAr contributes to synchrony of spontaneous activity in FCs and could be possible pharmaceutical target to future treatments aimed to alleviate tinnitus.

#### Differential Changes of the FC RLF Maximum Amplitude

The FC RLFs were significantly altered following the NMDAr antagonist with about half of the units (52%) showing an increase in their maximum amplitude while the other half (48%) showing a decrease (Figure 5). These two groups were differentiated by their RLF thresholds, i.e., units showing a decrease of their RLF maximum amplitudes had higher thresholds. This suggests that the NMDAr might provide a flexible mechanism to encode a wide dynamic range of FCs output relayed to the next auditory stations. The ANFs innervating the cochlear inner hair cells are characterized by low spontaneous rates with high thresholds and high spontaneous rates with low thresholds, respectively (Liberman, 1978). Recent research suggests a particular vulnerability of the high threshold fibers in “hidden hearing loss”, a pathology characterized by cochlear neural degeneration without hair cell loss, in humans and animal models with normal auditory thresholds (Kujawa and Liberman, 2009; Schaette and McAlpine, 2011; Furman et al., 2013; Viana et al., 2015). The FCs receive input from both low and high threshold ANFs (Liberman, 1993) but a relationship between their RLF thresholds and spontaneous rates has not been described. Our results suggest that the NMDAr could mediate such a relationship by differentially modulating the RLF maximum amplitude depending on the FC threshold.

### Blocking NMDArs Mediates Alterations of FCs StTDP

#### Changing the LRs

Three patterns of change were identified in StTDP of the FCs: units with initial Hebbian LRs transitioned towards anti-Hebbian and suppressive profiles while units with initial anti-Hebbian LRs transitioned towards Hebbian profiles (Figure 6). Pause-buildup and buildup units were more likely to change their LRs. Specifically, buildup units exhibited more transitions from Hebbian to any other profile while pause build up units showed more transitions from anti-Hebbian to any other profile (Figure 7). Previous studies indicate that the distinct fusiform temporal patterns of pause or buildup are determined largely by the fast inactivating A-type K^+^ current (Kanold and Manis, 1999). More recently, additional synaptic mechanisms were shown to contribute to the temporal response diversity of the DCN FCs. In particular, pause buildup units receive a stronger fast-rising excitation than buildup cells (Zhou et al., 2015). Interestingly, computational studies indicate that backpropagation of action potentials in the dendritic tree and non-linear amplification of the synaptic currents, two necessary conditions for STDP induction, depend on the A-type K^+^ channel distribution (Golding et al., 2001; Urakubo et al., 2004). It is therefore possible that the NMDAr interacts with both the intrinsic potassium channel conductance as well as with the fast-rising excitatory current to shape the plasticity of the FC responses in a unit type specific manner.

What would be the functional implications of unit specific transitions in the StTDP LRs? Studies in the hippocampus suggest that GABA-ergic inhibition can induce transitions from asymmetrical LR profiles to symmetrical, suppressive profiles (Cutsuridis, 2011). In the DCN, both the CWC and superficial stellate cells co-package glycine and GABA in the same vesicles (Apostolides and Trussell, 2013, 2014). Although the transmission appears to be predominantly glycinergic (Golding and Oertel, 1997; Mancilla and Manis, 2009; Apostolides and Trussell, 2013), GABA-ergic inhibition can significantly decrease the strength of inhibition (Davis and Young, 2000). Thus, it is possible that in FCs, NMDArs activation interacts with GABA-ergic inhibition to meditate the transitions from Hebbian to suppressive LR profiles observed in FCs (Figure 6, middle panel).

Furthermore, in cortical pyramidal neurons, the type of LR was shown to depend on the synapse location on the dendritic tree, with Hebbian LRs more likely to occur in the synapses closer to the soma and anti-Hebbian LRs in the more distal synapses (Froemke et al., 2005; Letzkus et al., 2006). This distribution is believed to facilitate a balanced contribution of the distal and proximal synapses to the probability of spike generation (Rumsey and Abbott, 2006). We showed that following NMDAr antagonist delivery one subgroup of FCs exhibited transitions from Hebbian to anti-Hebbian LRs (Figure 6, top panel) while a different group showed transitions from anti-Hebbian to Hebbian LRs profile (Figure 6, lower panel). These transitions therefore, might suggest a role of the NMDAr in controlling whether a distal or proximal group of synapses dominate the input and consequently the responses of the FCs.

#### Preserving the LRs

In DCN, we found that 30% of the FCs preserved their LRs (19% Heb, 8% aHeb and 3% Sup) following NMDAr antagonist delivery, indicating a form of plasticity that is less dependent on the NMDAr (Figure 8A). These units instead, showed a change in their plasticity to UA responses from a depressing to an enhancing profile (Figure 8B). This plasticity is unlikely to be a consequence of LTP/D induced changes since the ANF-FC synapse lacks this form of plasticity (Fujino and Oertel, 2003). Instead these changes are more likely to be mediated by the interactions between NMDAr and metabotropic GABAb receptors. GABAb is robustly expressed in DCN, primarily in the basal and apical dendritic tree of the FCs (Lujan et al., 2004; Salloum et al., 2014). *In vitro* studies have shown that in normal conditions, activating GABAb receptors with baclofen prevents the short-term depression of ANF-FC synapses and enhances the facilitation of the PF-FC synapses (Irie and Ohmori, 2008). However, prolonged activation of the NMDAr leads to endocytosis and subsequent lysosomal degradation of the GABAb receptors (Guetg et al., 2010; Terunuma et al., 2010), thus leading to a possible depression of ANF-FC synapses and an unchanged facilitation of the PF-FC synapses. Plasticity of FC synapses is then likely to drive mirror changes in the intrinsic degree of FC excitability and consequently in the FC *in vivo* tone-evoked responses (Debanne and Poo, 2010; Doiron et al., 2011). Indeed, studies *in vivo* showed that iontophoretic application of baclofen in DCN leads to a reduction of tone-evoked FC activity (Caspary et al., 1984). Collectively, these results suggest that when the NMDAr is active, *in vivo* UA stimulation of ANF-FC synapses expressing GABAbr (Juiz et al., 1994; Jamal et al., 2011) would result in a LTD while unimodal activation of the PF-FC synapses would result in a long term enhancement of FC responses. When the NMDAr is blocked, both UA and Sp5 stimulations would result in a long-term enhancement of the FC tone-evoked responses with no significant differences between the changes observed following SP5 unimodal stimulation evaluated before and after NDMAr antagonist, which is consistent with our observations (Figure 8C). While the interactions between the NMDAr and GABAbr depend on a site specific phosphorylation of the GABAbr (Guetg et al., 2010), and have differential effects on different GABAbr types (Kantamneni et al., 2014) it is less known whether certain NMDAr unit types are more efficient in mediating this function. Thus, it is possible that the FCs that preserve their StTDP LRs, but show robust auditory unimodal changes in plasticity express a type of NMDAr less sensitive to StTDP, but which interacts more robustly with GABAbr at the ANF-FC synapses.

In the present study, these units also showed a positive correlation between their spontaneous rates and the coefficient of variation. In general, a lower CV value indicates a more regular spiking pattern while a higher CV value indicates a more stochastic spiking activity (CV = 1 for a Poisson spike distribution). Interestingly, an increased degree of randomness of the neurotransmitter release at ANF synapses on ventral CN bushy cells increased the dynamic range of the synapse but decreased the spike-timing precision. This suggests that the degree of input randomness relates to whether spike-timing or spike distribution is the modality used for encoding information (Yang and Xu-Friedman, 2013). In this context, the direct relationship between spontaneous firing rate and the CV observed in the units preserving their LRs could indicate that these cells are more involved in temporal processing of auditory patterns and contribute less to DCN plasticity.

Previous studies (Koehler and Shore, 2013a,b) have shown that in the FCs of the normal hearing guinea pigs, the plasticity induced by BM is more robust than the one induced by unimodal auditory stimulation. However, in the present study, application of CPP caused unimodal auditory stimulation to have a greater long term effect in the LR-preserving units (Figure 9). This suggests that NMDArs are involved in the differential effects of unimodal and BM on long term plasticity.

### Influence of the Anesthetic

Systemic administration of a combination of ketamine and xylazine was used in the current study to induce and sustain the anesthesia. This anesthetic compound is broadly used in animal studies (Carter and Story, 2013), thus providing a critically important consistency of the results reported by various labs, which is instrumental to understanding the mechanisms of neural activity. However, ketamine is also a well-established NMDAr blocker (Liu et al., 2001). Therefore, it is important to understand whether its use as an anesthetic would have a significant impact on the results presented in this study.

Ketamine and CPP have different binding sites and mechanisms of action on the NMDAr. Ketamine is a non-competitive antagonist binding to a site located in the channel’s pore (Orser et al., 1997) while CPP is a potent competitive NMDAr antagonist binding at the glutamate site of the receptor (Harris et al., 1986). While the kinetics of the individual interactions between each antagonist with the NMDAr is well established by *in vitro* studies, less is known about their *in vivo* interaction. Some evidence suggests that CPP partially inhibits the ketamine binding site with a maximum level of 69% (Murray et al., 2000). Therefore, it is reasonable to conceive that the changes in the fusiform cell activity and plasticity reported in this study are mainly due to the effects of CPP antagonism and they could be an underestimate of the effects that could be seen when a different anesthetic would be used.

In conclusion, in this study we have identified several important contributions of the NMDAr to FC activity and StTDP. Blocking the NMDAr decreases synchronization of FC spontaneous activity and has differential effects on the RLF responses of low and high threshold units, which are more likely to increase and decrease, respectively, their RLF maximum amplitudes. Blocking the NMDAr alters the StTDP LRs by inducing transitions from Hebbian to anti-Hebbian and suppressive profiles and from anti-Hebbian to Hebbian profiles. We propose that these transitions reflect the contribution of the NMDAr to gating the importance of distal and proximal dendritic synapses to the FC responses with possible influences on the dynamic filtering function of the DCN. A fraction of units showed plasticity that was less dependent on the NMDAr. It is possible these units are more involved in precise temporal processing of acoustic stimuli than alterations due to long term synaptic plasticity.

## Conflict of Interest Statement

The authors declare that the research was conducted in the absence of any commercial or financial relationships that could be construed as a potential conflict of interest.
